# Ablative techniques for the treatment of benign and malignant breast tumours

**DOI:** 10.1186/s40349-017-0097-8

**Published:** 2017-07-03

**Authors:** Mirjam C. L. Peek, Michael Douek

**Affiliations:** Division of Cancer Studies, King’s College London, Guy’s Hospital Campus, Great Maze Pond, London, SE1 9RT UK

**Keywords:** Breast, High intensity focused ultrasound (HIFU), Radiofrequency ablation (RFA), Cryo-ablation, Laser ablation, Microwave ablation

## Abstract

Minimally invasive techniques like high intensity focused ultrasound, radiofrequency ablation, cryo-ablation, laser ablation and microwave ablation have been used to treat both breast fibroadenomata and breast cancer as an alternative to surgical excision, potentially reducing the complications, improving cosmesis and reducing hospital stay. This review describes the most common minimally invasive techniques available, their history and some of the studies performed with these techniques in both benign and malignant lesions. In addition we described some of the difficulties of using these minimally invasive techniques such as optimization of anaesthesia, imaging and immobilisation in order to increase the complete histopathological ablation rates.

## Background

Breast conditions like benign fibroadenomata and breast cancer are common and thus of public interest. About one in eight women will be diagnosed with breast cancer in their lifetime and about 50% of all breast biopsies are for a breast fibroadenoma [1, 2]. The incidence of breast lumps has increased due to the raised awareness of breast cancer and more women performing breast self-examination. Furthermore, screening programs are more widespread and improved in quality, reaching more women. Breast cancer screening programs and general practitioners with patients with breast lumps refer women to one-stop Breast Clinics. Diagnosis is made using triple assessment; history and physical examination, imaging with either ultrasound (US) and or mammography and pathological assessment with either aspiration cytology or core biopsy [1, 3]. For breast fibroadenomata there are currently three treatment options, patients with non-symptomatic lumps are generally reassured that the lump is benign and does not require treatment. Patients with symptomatic fibroadenomata are reassured, but can request treatment; either vacuum assisted mammatomy or surgical excision. For patients with early diagnosed breast cancer, the standard treatment consists of breast conserving surgery and sentinel lymph node biopsy [4–6]. In addition, patients may require further adjuvant treatment in the form of chemotherapy, radiotherapy or hormonal treatment. If the breast cancer is more advanced, mastectomy and sentinel lymph node biopsy or axillary node clearance are also performed when nodes are involved.

Recent developments include several new techniques to improve the treatment of both benign and malignant breast tumours. Techniques like radiofrequency ablation (RFA) and high intensity focused ultrasound (HIFU) have been developed in order to provide lesser invasive alternative techniques and improve cosmesis. Cosmesis has become more important for both patients and surgeons and operations have become lesser invasive with tissue preservation, displacement or replacement being implemented more often [7]. Minimally and non-invasive treatments cause less complications, a shorter hospital stay and are potentially cheaper [8, 9]. However, there are some disadvantages such as the absence of a tissue specimen after treatment to determine the final pathology, and retention of the tumour and tumour bed during treatment, which may be disconcerting for some patients. In addition, surgical resection is not an option for all patients and ablative techniques can be used in patients with co-morbidities or poor health [10]. This review describes the different ablative techniques available and the evidence supporting their use the treatment of breast tumours. A systematic search was carried out using the search method described in a recently published systematic review [9] and this search was updated on January 31st 2017.

### High intensity focused ultrasound (HIFU)

HIFU is a completely non-invasive ablative technique in which a focussed US beam propagates through tissue as a high-frequency pressure wave causing the temperature to rise, leading to protein denaturation and coagulative necrosis. The surrounding tissues are not damaged and no incision is required. HIFU can be guided with either US or MRI [11, 12]. Currently, HIFU has been successfully used in the treatment of both benign and malignant tumours in the liver, breast, kidney, uterine, prostate and pancreas, and in the treatment of osteosarcomas and as a pain relief in bone metastasis [12]. HIFU has also been used for neurological diseases but apart from essential tremor, tremor dominant Parkinson’s and neuropathic pain where there is CE marking, there are no other licenced indications at present. Other indications currently in development include depression, cerebral tumours, obsessive compulsive disorder, cortical excitation, mechanical cavitation and drug delivery across the blood brain barrier [13].

The first use of focused ultrasound was in 1942, however, the first report in humans was not until 1950 [14] when 50 patients with Parkinson’s disease were treated [12]. The first clinical application of focused ultrasound was for extracorporeal shockwave lithotripsy as a method for treating kidney stones. Focused ultrasound was re-discovered in 1990, due to the development of modern technology and advanced imaging methods and the realization that focused ultrasound can produce instant cell death [15]. The first study using HIFU for breast tumours was performed by Hynynen et al. [16] who used magnetic resonance guidance (MRI) guided HIFU on 11 patients with fibroadenomata. The HIFU system used included a transducer with a focal length of 100 mm in diameter, 1.5 MHz frequency and 1.5 T scanner (ExAblate, GE healthcare, USA). Complete or partial ablation was achieved in eight patients and no adverse events were observed. Gianfelice et al. [17–19] performed three studies treating 12, 17 and 24 patients with MRI guided HIFU using a transducer with a focal length diameter of 100 mm, a frequency of 1.5 MHz and a 1.5 T MRI (ExAblate 2000, GE healthcare, USA and InSightec-TxSonics Ltd, Israel). Histopathological complete ablation was found in 17%, 24% and 79% respectively and skin burns were observed in two patients in the first study and one patient in the last study. Furusawa et al. [8, 20] performed two studies on 28 and 21 patients respectively with MRI guided HIFU and found a complete ablation rate of 53.5% in the first study were the tumours were excised after five to 23 days and in the second study, no complete ablation rates were described and patients were followed-up for 14 months (3 to 26 months). Both studies used a 1.5 T MRI ad ExAblate 2000 device (InSightec-TxSonics Ltd, Israel; no characteristics reported). The first study reported one patient with a skin burn and in the second study, two skin burns were observed and one patient had a recurrence. Peek et al. [21] performed a study on 20 patients and 20 control patients with US guided HIFU and found a significant reduction in size of 43.5% (*p* = 0.016) after six months compared to a non-significant 4.6% (*p* = 0.530) for the control group demonstrating a significant difference between the two groups (*p* = 0.002), thereby rejecting the current belief that these lumps regress spontaneously. This study used the Echopulse device (Theraclion, France) with an imaging transducer of 7.5–12 MHz and treatment transducer of 56 mm in diameter and a frequency of 3.0 MHz. Merckel et al. [22] included ten patients with early stage invasive breast cancer and used an MRI guided HIFU system (Sonalleve, Philips Healthcare, Finland) with a frequency of 1.45 MHz, 32 transducer elements with a diameter of 6.6 mm and an integrated 1.5 T scanner. Tumour necrosis was observed with a maximum diameter of 3–11 mm and no complications were reported. No correlation was found between the temperature increase and the applied energy but there was a good correlation visible between the applied energy and the size of necrosis seen within the tumour.

### Radiofrequency ablation (RFA)

Radiofrequency ablation (RFA) can be used for both curative and palliative indicators and has been used for solid tumour throughout the body including liver tumours, lung nodules, kidney tumours, bone tumours, bone metastases and breast cancer. It uses low frequency radio-waves with a long wavelength to generate heat and cause coagulative necrosis. During RFA, a needle electrode is inserted percutaneously under US guidance to deliver an alternating current that generates ionic agitation, localised tissue heating and cell death. RFA differs from other techniques as the heat is not supplied by the probe itself, however this will lead to a limiting volume which can be ablated at a time and multiple probes are required for large lesions. RFA can be used with US, computed tomography (CT) or MRI and can be used percutaneously or during surgery [10].

RFA was first used in the 1900s by William Bovie and not much has changed in the technique since the first study. The technique has been used for almost a century for superficial and easy to reach tumours and lesions. In the meantime, the electrical scalpel was developed which is a form of RFA. Due to the development of imaging systems with an increased sensitivity and specificity and improved image guided modalities the RFA technique became available to treat more difficult to reach areas, and increasing the power output, larger areas could be ablated with a single probe [23].

One of the first studies was performed by Izzo et al. [24] who performed a treat and immediate resect feasibility study in 26 patients with a mean tumour size of 1.8 cm (0.7–3.0 cm). Treatment was performed under US guidance using a LeVeen needle electrode (Radiotherapeutics, Corporation, USA) by using a power of 10 watts for two minutes, followed by an increase in steps of five watts per minute until tissue impedance increased and the power dropped to below 10 watts. Complete pathological ablation was obtained in 96% of all patients and side effects were seen in one patient who developed a skin burn. This study was followed by many other studies including the study by Oura et al. [25] who treated 52 patients with a mean tumour size of 1.3 cm (0.5–2.0 cm). Treatment was performed using a CoolTip radiofrequency needle (Valleylab, USA) under US guidance and started with five watts for one minute then 10 watts for one minute followed by an increase of 10 watts per minute until the generator stopped working due to an increase in impedance of more than 20 ohms or due to treatment time reaching 30 min. Complete ablation was found in 100% of patients following core needle biopsies, with only one patient with a skin burn as a complication. Patients were followed up for 15 months (6 to 30 months). Manenti et al. [26] performed a study in 34 patients with tumour with a mean size of 1.9 cm (1.7–2.0 cm). Treatment was performed using the CoolTip system (Miras PTV, Italy) under US guidance with an initial power according to the patients initial impedance for five minutes and this power was increased by 10 watts every minute thereafter until the target temperature of 90° Celsius was reached. Next the power was adjusted to maintain the target temperature for 12 min and this was followed by a cool-down period of one minute. A 97% pathological complete ablation was found and tumours were resected after four weeks and one patient developed a skin burn. Kinoshita et al. [27] included 49 patients with mean tumours 1.7 cm (0.5–3.0 cm) in size and found a complete ablation rate of 61% on histopathology, after immediate resection and reported two skin burns and three muscle burns. The CoolTip system (Covidien, Energy-Based Devices, USA) was used under US guidance and an initial power of 10 watts was used which was increased by five watts every minute until the maximum power of 55 watts was reached. More recently, Manenti et al. [28] performed a comparison study including 40 patients who underwent RFA and 40 patients who underwent cryo-ablation and patients underwent surgery 34 days (30 to 45 days) after treatment. Complete ablation was found in 92.5% of the RFA group and 95.0% for cryo-ablation. No complications were described. The RFA ablation system used was the CoolTip needle (Miras PTV, Italy) under US guidance using the same protocol as described before.

### Cryo-ablation

With cryo-ablation, a probe is inserted into the tumour under US guidance and is the only technique which uses freezing instead of heat to create tumour necrosis. The ablation process involves two phases: freezing and thawing, comprising of four mechanisms to destroy tumour cells: direct damage by intracellular ice formation and osmotic dehydration and indirect damage due to ischaemia and immunologic response. Cryo-ablation is the only technique which utilizes cold thermal energy and most often a protocol of two 10 min cycles of freezing is used with a five to 10 min break of thawing. The skin requires protection from the low temperatures but the pectoralis major muscle is less sensitive to temperature change than breast tissue. Cryo-ablation is most commonly used for open and laparoscopic surgery but the application for tumour ablation is becoming more popular. The technique can be guided by either US, MRI or CT [29].

Cryo-ablation was first used in 1845 by James Arnott when advanced cancers were frozen using iced salt solutions, resulting in a reduction of the tumour size. In the 1900s, other methodologies such as solid carbon dioxide, liquid nitrogen and nitrous oxide became available which further developed the technique. However, only superficial small lesions could be treated. The system as it is now was created by Cooper et al. in 1961, and was used for the treatment of visceral cancer. Due to improvements in the device and imaging techniques in the late 1980 the technique was further developed to the technology used at present [30].

The first study describing cryo-ablation for the breast was from Pfleiderer et al. [31] and included 15 patients with a mean tumour diameter of 2.1 cm. The procedure was performed using a 3 mm CryoHit probe (Galil, Israel) under US guidance by freezing for 7–10 min, thawing for five minutes, freezing again for 7–10 min and thawing until the ice-ball was melted. Patients underwent surgery within five days of treatment. No complications were reported, patients with tumours under 1.6 cm were found to have complete ablation, however the 11 patients with tumours up to 2.3 cm had sections of incomplete ablation. Pfleiderer et al. [32] performed a second study on 30 patients with a median diameter of 1.2 cm. The same system and procedure was used as in the first study and patients underwent surgery within six weeks. In 24 of 30 patients (80%), complete ablation was achieved whilst in one patient the cryo-procedure was not completed due to technical problems. Simmons et al. [33] performed a study on 86 patients (87 tumours) with a mean diameter of 1.2 cm. Treatment was performed using the Visica system (no details reported). Complete pathological ablation was achieved in 75.9% of patients and the negative predictive value on MRI was 81.2%.

Nurko and Edwards et al. [34, 35] performed a study on 444 patients with a mean fibroadenomata size of 1.8 cm and showed a reduction in size of 51% after six months. The Visica treatment sytem (Sanarus Medical, USA) was used with a 2.7 mm cryo probe under US guidance and using two cycles of freezing (first high then low setting to maintain ice ball) and thawing. In addition, palpability of the fibroadenomata reduced from 75% at pre-treatment to 46% at six months and 35% at one year. Patients were satisfied with the treatment in 91% of cases after six months and 88% of cases after one year.

### Laser ablation

With laser ablation, tissue is destroyed by direct heating with low-power and high intensity laser light energy delivered percutaneously via thin optical fibres. Upon absorption of the tissue, heat is produced, inducing lethal thermal injury. There are three types of lasers available; the carbon-dioxide laser, argon laser and Nd-YAG laser. The latter two have the benefit of being able to treat larger volumes due to lower absorption coefficients and the ability to be used within single fibres, making it possible to use them in combination with flexible endoscopes [36, 37]. Imaging guidance during treatment is performed with either US, CT or MRI.

The first use of a laser was described in 1960 and not long afterwards laser ablation was first described in 1983 when a laser consisting of a light conducting quartz fibre was used to ablate tumour tissue. However, due to the technical difficulties of using the device and targeting the correct area, the technique lost interest. The development of more sophisticated and reliable lasers and use of more flexible and better quality guiding systems has resulted in a gained interest and opened up a number of applications for laser ablation [36].

Harries et al. [38] performed the first laser ablation study in 1994 including 44 patients. Treatment was performed under US guidance in 42 patients and CT guidance in two patients using a semiconductor laser (Diomed, UK) with a wavelength of 805 nm and a 400 μm fibre. The tumour was treated for 500–750 s with a power of 2–3 watts. Necrosis within the excised tumour varies from 0 to 2.5 cm and excision was performed after one to 34 days. US was found to be inaccurate in determining the extent of tumour necrosis, whereas CT and MRI were more effective. Dowlatshahi et al. [39–41] performed a study on 54 patients with a mean diameter of 1.3 cm. A semiconductor laser diode with an 805 nm wavelength (Diomed-25, Diomed, UK) was used under US guidance with a laser fibre of 400–600 μm. Treatment was started at five watts until the sensors at the periphery measured a temperature of 60° Celsius. Complete pathological ablation was found in 70% and two patients suffered a skin burn. The tumours were excised after one to eight weeks of treatment. More recently van Esser et al. [42] included 14 patients with a mean diameter of 1.7 cm and excision was performed straight after laser ablation. An Nd:YAG continuous laser (KLS Martin, Germany) of 1045 nm was used with a 25 mm Microdom LITT laser fibre (no procedure description reported). Single cases of a skin burn and a pneumothorax were reported as complications. Complete pathological ablation was obtained in 50% of patients; in 88% of tumours smaller than 2.0 cm and in 17% of tumours of ≥2.0 cm. Lai et al. [43] performed a study on 24 patients with fibroadenomata with a mean size of 2.5 cm (1.4–3.5 cm) and showed a reduction of 60% after six months. Treatment was performed under US guidance with Diomed-25 (Diomed, UK) with a wavelength of 805 nm and a power of 2.5 watts per fibre for a total duration of 500 s.

### Microwave ablation

Microwave ablation uses electromagnetic waves to induce tumour destruction using devices capable of delivering frequencies of at least 900 MHz. It uses localised heating caused by water molecules which move within tissues, and externally applied focussed microwaves to cause tissue necrosis. This technique can heat and damage high-water-content tumour cells, while tissues with lower-water-content such as adipose and breast glandular tissues remain unharmed. It is similar to RFA but offers a more consistent high temperature, larger tumour ablation volumes, faster treatment times and lower treatment pain scores. The technique can be guided by either US or CT and the system by Vivant Medical (Mountain View, Calif) is the only system available for humans in America [44].

Microwave ablation has not been used for fibroadenomata, only for breast cancer ablation. Gardner et al. [45] was the first to publish about this technique and included ten patients with a mean tumour size of 4.3 cm. An E-field feedback probe was used to focus the microwaves along with a temperature sensor catheter. Treatment was performed under US guidance and completed when the desired treatment dose was reached or when the treatment time reached 40 min. The mean maximum temperature was 44.9° Celsius and the treatment time was 34.7 min. On US a significant reduction in tumour size was seen in 60% and a significant tumour response was seen in 80% of patients. Complications included flap necrosis in three patients and a blister in one patient. Dooley et al. [46] performed a series of studies on microwave ablation using a focused microwave thermotherapy system (model APA-1000, Medifocus, Inc, Canada) with 915 MHz waveguide applicators and E-field focusing sensors under US guidance. It was shown in a phase I study including ten patients that a single low dose had a partial tumour response; in a phase II study the thermal dose to obtain 100% pathological response was determined. In the first randomised controlled trial (RCT) it was shown that patients with early breast cancer who received breast conserving surgery with microwave ablation had 0% (0 of 34) positive tumour margins whilst those who only had breast conserving surgery had 9.8% positive tumour margins (4 of 41). The second RCT showed that for patients with large tumours, neo-adjuvant chemotherapy in combination with microwave ablation showed a reduction of 88.4% (*n* = 14) on US whilst neo-adjuvant chemotherapy alone showed a reduction of 58.8% (*n* = 10). More recently, Zhou et al. [47] performed two studies including 41 patients with a mean tumour volume of 2.0 cm. The system used was designed by the research group and used an irradiation frequency of 2450 MHz and an output power of 40 Watts. Complete pathological tumour ablation was seen in 90% (37 of 41) of patients and the only complications were skin injury in one patient and pectoralis muscle injuries in two patients. The second study included 12 patients with a tumour size of 2.89 cm and showed the tumour decreasing in size on MRI.

## Discussion

The lack of RCTs, the rapid change in practice, different techniques evaluated and wide variations in techniques between hospital sites make it difficult to assess the potential role of these techniques in the treatment of breast disease.

In general, all ablative techniques show a variety of results in terms of complete ablation, complications and treatment times, making it difficult to analyse results and reach conclusions. More studies using identical technique protocols are required in order to improve the data (obtain complete pathological ablation in all patients), standardise the techniques and in order to obtain the best ablative technique in each field. In addition, comparative studies are required to then compare ablative techniques to each other to decide which of them is most promising. Lastly, the most promising technique should be compared to the current standard of care and a cost-effectiveness analysis is required.

The lack of complete histopathological ablation can be due to several factors, a lack of immobilisation or a lack of appropriate anaesthesia resulting in patient movement and an inefficient treatment or a wrong choice in imaging guidance.

Immobilisation is required in order to minimize movements caused by discomfort due to the position of the patient, breathing, pain caused by the treatment or other body movements. It is therefore important to make sure there is an immobilisation system available which reduces movement without affecting the treatment. Movement by the patient can also be reduced by increasing the level of anaesthesia given to the patient. Due to the increase of complications, hospital stay and the requirement of an anaesthetist, general anaesthesia is not recommended. However, simple local anaesthesia may not be effective enough in order to be able to use the technique optimally. Therefore other sedative options such as a pectoralis major block, conscious sedation or a combination of local anaesthetics may prove to be better.

The type of image guidance is very important, MRI provides a good resolution and the ability of temperature mapping, however it is expensive, labour intensive and not mobile. US provides real-time imaging, is cheap and it does not require extensive training to learn how to interpret the images. However, it cannot provide temperature mapping or provide information about the flow in the vessels. In addition, for needle insertions MRI guidance is more complicated as needles need to be MRI compatible, to avoid metal displacement and artefact. CT is able to provide a good spacial resolution but also cannot visualise the flow within vessels or temperature changes in real time. However CT is cheaper and more accessible than MRI. US guidance facilitates delivery of local anaesthesia, and provides easier access to the patient facilitating any adjustment to immobilisation. However, both patient movement and tissue displacement by injection of local anaesthesia, can affect US assessment of response to treament [11, 12, 48]. In terms of sedation and immobilisation, both CT and MRI require for the patient to be fully sedated.

For RFA, US is the preferred guidance technique as the 500 kHz RFA source can interfere with MRI and cause artefacts, making it difficult to follow treatment progression. MRI compatible probes have been developed but are expensive. On US, a coagulative opaque area is seen after treatment which corresponds to a macroscopically yellow-white area with a haemorrhagic rim and microscopically area of coagulative necrosis and protein denaturalisation [37, 49]. However, during treatment no tissue changes are visible [50]. RFA benefits from a short treatment time but patients require general anaesthesia, prolonging the total treatment time and increasing the complication rate. HIFU is preferred for smaller lesions and has a much longer treatment time compared to RFA. However, it is the only technique which requires no skin incisions, thereby reducing the wound related complication rate. In addition, on US a hyper echoic cross or mark is seen during and after treatment, indicating cavitation. Laser ablation has a relatively short treatment time and post-treatment concentric rings with a central cavity surrounded by a paler zone of viable and non-viable cells and a haemorrhagic rim are shown [37, 49]. Apart from being the only technique using cold instead of heat, cryo-ablation benefits from its analgesic properties, requiring reduced anaesthesia compared to other techniques. In addition, tissue changes during freezing can be seen on US but not on mammography post-treatment. Microscopically the outcome is similar to RFA and laser ablation with a central cavity of hyaline necrosis and a peripheral rim of tissue with viable cells [37, 49, 50]. The advantage of microwave ablation is its high thermal efficacy, but this is also a disadvantage as surrounding tissues are easily ablated as well. Additional advantages are a short treatment time and the absence of grounding pads which are used in RFA to prevent skin burns [51] (Table 1).

**Table 1:** Characteristics of ablative techniques

	Imaging guidance^a^	Appearance	Advantage	Disadvantage
High Intensity Focused Ultrasound	MRI, US	One system integrated with imaging guidance	Non-invasive	Time-consuming
Radiofrequency ablation	MRI, US	Needle connected to system	Time efficient	Minimally invasive
Cryo-ablation	US	Probe connected to system	Freezing instead of heating, analgesic effect	Time-consuming, minimally invasive
Laser ablation	US, CT	Fibre connected to system	Time efficient	Minimally invasive
Microwave ablation	US	Probe connected to system	Time efficient and high thermal efficacy	Minimally invasive, time consuming, more treatment related complications

^**a**^ *US* ultrasound, *MRI* magnetic resonance imaging and *CT* computed tomography

Finally, histological assessment for confirming response to treatment needs to be improved as several studies have shown that staining using haematoxylin and eosin is not sufficient in demonstrating cell death in ablated tissue. Whilst staining with nicotinamide adenine dinucleotide does confirm cell death, it needs to undergo quality assessment before it can be used routine histology. The major disadvantage of all ablative techniques is lack of histopathology and therefore it is important that prior to treatment, pathology is obtained on biopsy and patient’s are followed up with imaging. In addition, patients need to be well informed about the remaining lump after treatment and that their lump will gradually decrease in size.

## Conclusion

Minimally invasive techniques have been used to treat both benign and malignant breast lesions as an alternative to surgical excision but reduction in complications, improve cosmesis and reduced hospital stay, still require to be demonstrated. However in order to improve complete histopathological ablation rates and make it possible to set up comparative trials to determine the most promising technique, the existing techniques require optimization, improved anaesthesia, better imaging and better immobilization. 

## Abbreviations

CT: Computed tomography
FAD: Fibroadenomata
HIFU: High intensity focused ultrasound
MRI: Magnetic resonance imaging
RFA: Radiofrequency ablation
US: Ultrasound
